# High-Temperature Compressive Properties of 3D Printed Polymeric Lattice-Reinforced Cement-Based Materials

**DOI:** 10.3390/polym17060802

**Published:** 2025-03-18

**Authors:** Yawen Gu, Jing Qiao, Junwei Liu, Wenfeng Hao, Can Tang

**Affiliations:** 1 Lianyungang Technical College, Lianyungang 222000, China; 2 College of Mechanical Engineering, Yangzhou University, Yangzhou 225127, China; 3 Faculty of Civil Engineering and Mechanics, Jiangsu University, Zhenjiang 212013, China; 4 College of Civil Science and Engineering, Yangzhou University, Yangzhou 225127, China

**Keywords:** cement-based composites, 3D printing, compressive mechanical properties, digital image correlation (DIC), high temperature

## Abstract

Currently, research has confirmed the significant potential of 3D printed polymer lattices in enhancing the mechanical properties of cement-based composites. Polymer materials are influenced by high-temperature environments. This study aims to further explore the impact of 3D printed lattice structures on the compressive mechanical properties of cement-based materials under high-temperature conditions. The approach employed in this paper involves utilizing the multiple jet fusion (MJF) technique to fabricate two types of lattices with the same volume fraction within cement-based samples. Uniaxial compression experiments were carried out on cement-based samples both with and without the 3D printed lattice at room temperature, 50 °C, and 100 °C. The research explores the compressive properties of cement-based samples reinforced with different lattice structures at varying ambient temperatures. Additionally, digital image correlation (DIC) technology was utilized to analyze the deformation characteristics of the samples. The experimental results demonstrate that the 3D printed lattice effectively enhances the compressive properties of cement-based materials. However, it is worth noting that the cement-based samples reinforced with this material exhibit higher temperature sensitivity compared to the lattice-free reinforced samples.

## 1. Introduction

When exposed to the natural environment, building structures undergo testing from various environmental factors. During summer, high temperatures and intense solar radiation can significantly raise the surface temperature of concrete buildings. Consequently, temperature differences and temperature gradients induce thermal stresses within the structure, affecting both internal and external components. These stress-induced deformations can result in cracks, joint deformation, and other forms of damage, ultimately leading to structural failure. These damages not only impact the appearance and service life of the concrete structure [1,2,3,4,5], but more critically, they diminish the overall structural safety. Therefore, in-depth studies on the temperature effects caused by high-temperature environments are imperative to ensure the safety of concrete structures during service.

Scholars have made significant progress in investigating the influence of high-temperature environments on concrete performance. For instance, Zhao [6] and other researchers conducted physical experiments to reveal the cracking law of C60 high-strength concrete under varying heating temperatures (20 °C, 100 °C, 200 °C, 400 °C, 600 °C, 800 °C) and constant temperature durations. Their findings indicated that the main cause of macroscopic cracking in high-strength concrete is the decomposition occurring within the concrete due to high temperatures. This decomposition leads to gaps and small cracks within and on the surface of the concrete samples, subsequently affecting the overall mechanical properties of the structure. Akca [7] examined the residual properties of concrete structures after exposure to high-temperature environments (concrete specimens were heated to 1000 °C), analyzing the microstructure of concrete samples using XRD, TGA, and SEM techniques. Tao et al. [8] explored the deterioration mechanism of concrete under prolonged high temperatures, revealing that the concrete’s constituent materials decompose when exposed to high temperatures. This decomposition leads to the formation of cracks and holes, resulting in severe deterioration of the concrete structure. These studies not only establish a robust academic foundation but also provide valuable guidance for investigating the evolution of mechanical properties in 3D printed polymer lattice-reinforced cement-based composites under high-temperature conditions.

Currently, research has been conducted on the potential applications of 3D printed structures [9,10] in the realm of cement-based composites. Traditional reinforced concrete structures, when exposed to corrosive environments, are prone to cracking and spalling of the concrete cover, leading to corrosion of the steel reinforcement. This results in a sharp decline in the durability of the reinforced concrete structure, damages its integrity, and reduces its service life. FRP (Fiber-Reinforced Polymer) bars, as a new type of composite reinforcement, are considered an ideal substitute for steel reinforcement in corrosive environments. However, the brittle failure characteristics of FRP-reinforced concrete components and the issues of excessive crack width and deflection under normal service conditions limit their application. Three-dimensional printing (additive manufacturing) is an emerging technology developed in recent years, characterized by personalized manufacturing, fast printing speed, and the ability to print complex structures. This technology enables precise control over the distribution of fibers within concrete. Whether 3D printed grids can partially or entirely replace steel reinforcement in the future, similar to FRP bars, addressing the durability shortcomings of reinforced concrete, improving its mechanical properties, and forming a new structural type, is a topic worthy of in-depth research. Farina [11] utilized 3D printing technology to fabricate polymer and metal reinforcement structures with different surface morphologies and roughness, aiming to enhance cement mortar. Through a detailed investigation using a three-point bending test, they examined the influence of surface roughness on the bond strength between steel bars and cement mortar. Nam et al. [12] employed 3D printing technology to design a model capable of precisely controlling the location and orientation of fiber distribution within concrete. They conducted three-point bending tests to analyze the reinforcement effect of fiber distribution in different spatial arrangements on the flexural mechanical properties of cement mortar. Xu et al. [13] confirmed the great potential of using additive manufacturing to create cement-based composites with customizable properties. Junaid et al. [14] evaluated the bending properties and ductility of small mortar beams reinforced with 3D printed polymers, observing significant improvements in strength and ductility with the use of most thermoplastics. Hao et al. [15] employed selective laser sintering (SLS) to construct three-dimensional lattice structures and analyzed the mechanical behavior and failure characteristics of polyamide12 (PA12) fiber-reinforced composite lattice structures using digital image correlation (DIC) methods. Xu et al. [16] investigated the reinforcement effects of 3D printed polymer networks with different structures on the mechanical properties of cemented composites.

Presently, research on the mechanical properties of 3D printed polymer lattice-reinforced cement-based composites mainly focuses on evaluating mechanical properties at room temperature, with limited exploration of their mechanical behavior and deformation characteristics at high temperatures. According to Reference [17], studies have investigated the effects of 3D printed cell structures with various forms on the compressive mechanical properties of cement-based composites. These cell structures include circular, cubic, kelvin, octagonal (Oct), rhombic truncated dodecahedron (RO), and enhanced octagonal (SO) structures. The experimental results indicate that cell structures with different forms exhibit distinct edge deformation mechanisms during the stress process.

To further investigate the specific effects of a high-temperature environment on the mechanical properties of cement-based composites under uniaxial compression, this study focuses on two lattice structures (RO lattice and SO lattice) that exhibit significant mechanical enhancement effects on cement-based materials. Cement-based materials without lattice reinforcement are used as the control group. Uniaxial compression experiments are carried out at room temperature, 50 °C, and 100 °C after 28 days of standard curing.

## 2. Experimental Details

This study utilizes a lattice design that incorporates the control variable method, aiming to maintain an equivalent volume fraction of two distinct structural forms, namely SO and RO, within the cement-based material. A comparison is made against the control group, which consists of pure cement-based composite material without 3D printing lattice reinforcement. The objective is to investigate the effect of high temperature on the mechanical properties of 3D printed lattice-reinforced cement-based composites under a uniaxial compression load.

### 2.1. Three-Dimensional Printed Lattice Design

The 3D printed lattice sample employed in this study was fabricated using multi-jet fusion (MJF) technology [18,19] and belonged to the material group known as nylon 6 (PA6) [20,21,22,23,24]. Shalchy et al. [25] conducted a quantitative study on the adhesion energy between various polymers and cement matrix, revealing that PA6 exhibited the highest binding force. This material possesses desirable characteristics such as lightweight nature, high strength, good thermoplasticity, excellent mechanical properties, superior wear resistance, good toughness, and ease of processing. In addition, it exhibits favorable resistance to solvents and corrosion.

During the design phase of the 3D printed lattice, computer-aided design (CAD) software was utilized to construct a 3D model. As illustrated in Table 1, each cell’s central line was uniformly set at dimensions of 10 mm × 10 mm × 10 mm and geometrically arranged around three intersecting vertical planes centered on the cell. This arrangement resulted in a complete lattice structure comprising 4 × 4 × 4 cells. Simultaneously, the volume fraction of the lattice structure, strictly controlled by the pillar diameter, accounted for 8% of the total sample volume. Leveraging the CAD modeling’s information query function, a cubic polynomial function was employed to describe the relationship between the diameter and volume of the lattice pillar. The relationship between the diameter variable and the lattice’s volume (V) was fitted using the following cubic polynomial equation:

$$V_{RO}=f(d_{RO})=-2\times 10^{-5}d_{5}+8115.9d_{5}^{2}-1151.5d_{5}^{3}$$

$$V_{SO}=f(d_{SO})=0.0003d_{6}+7072.3d_{6}^{2}-900.55d_{6}^{3}$$

### 2.2. Cement Base Material

**Figure 1:**
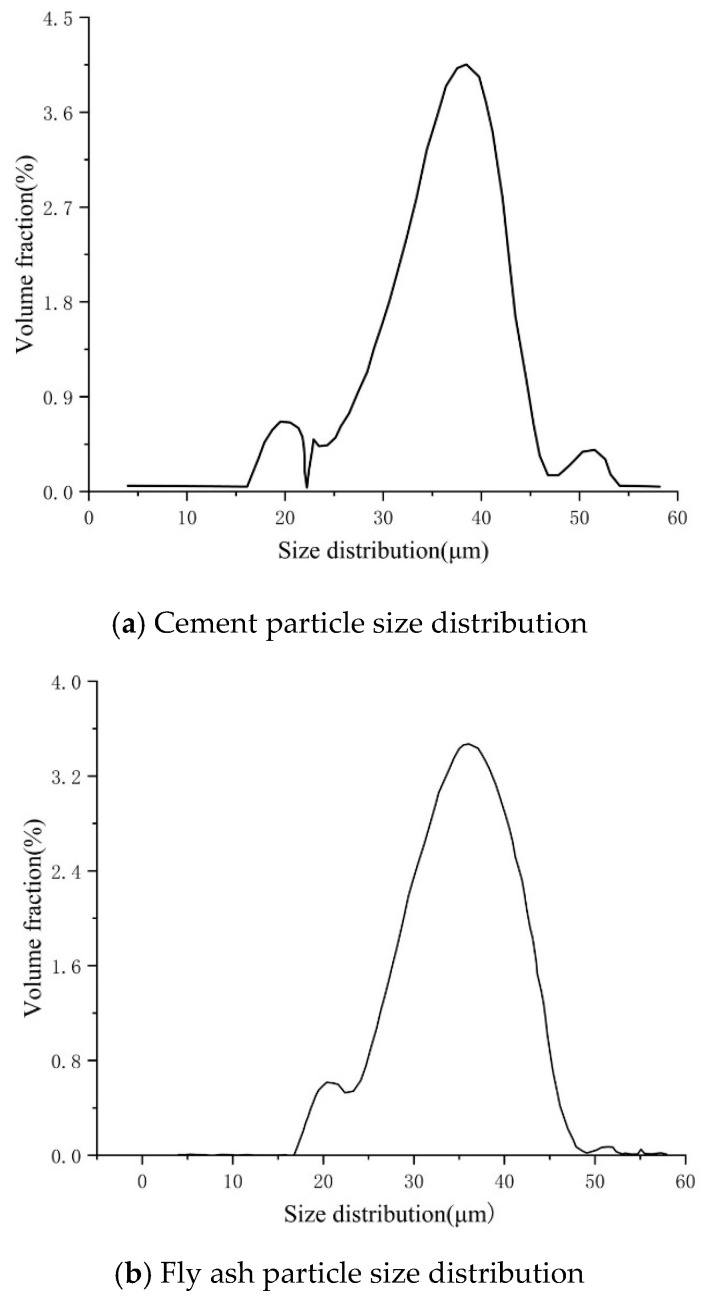
Particle size distribution.

The raw materials employed in the preparation of cement-based materials for this experiment include cement, fly ash, standard sand, water-reducing agents, and water. The key performance indicators of these raw materials are outlined below:

1. Water: Zhenjiang tap water was utilized.
2. Cement: Ordinary Portland cement PO42.5, produced by Jiangsu Hailin Cement Co., LTD. (Taizhou, China), was used. The main mineral composition of this cement is presented in Table 2. The particle size distribution curve of cement particles is depicted in Figure 1a, with particle sizes concentrated between 3 and 60 μm and an intermediate particle size of 19.9 μm.
3. Fly ash: The first-class fly ash produced by Gongyi Bairun Refractory Co., LTD. (Gongyi, China). The particle size distribution of fly ash is shown in Figure 1b.
4. Water-reducing agent: Polycarboxylic acid water-reducing agent produced by FOkker Technology Co., LTD. (Suzhou, China). The main performance indicators are summarized in Table 3.
5. Standard sand: Chinese ISO standard sand produced by Xiamen ISO Standard Sand Co., LTD. (Xiamen, China) (particle size range: 0.08 mm to 2 mm). Its main mineral composition is tabulated in Table 4.

**Figure 2:**
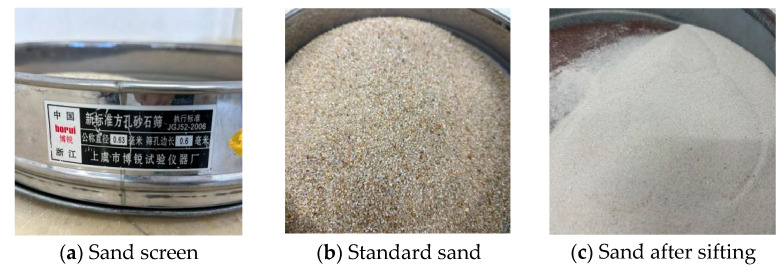
Standard sand sifting.

To accommodate the inclusion of the 3D printed lattice in the experiment’s sample, it was necessary to ensure adequate fluidity of the cement mortar. To achieve this, the standard sand underwent screening, as depicted in Figure 2. A new standard square hole sand screen, produced by Shangyu Borui Test Instrument Factory (Shaoxing, China), was utilized. This screen possesses a nominal diameter of 0.63 mm and a side length of 0.6 mm.

### 2.3. Cement-Based Composite Material Mix Ratio

The mix ratio of the cement-based composite materials prepared using the aforementioned raw materials is presented in Table 5. The dimensions of the cement-based sample were set at 42 mm × 42 mm × 42 mm.

### 2.4. Specimen Preparation

The production of cement-based samples involves several steps, including weighing, sampling, feeding, mixing, grouting, pouring, vibration, compaction, molding, and maintenance. To create the lattice-reinforced cement composite structure, a casting mold was utilized. The samples were demolded after 24 h and subsequently cured in a standard curing box for a duration of 28 days. There are 3 samples for each test condition and 9 samples for each temperature (room temperature, 50 °C, 100 °C), for a total of 27 samples.

### 2.5. Experimental Facilities

**Figure 3:**
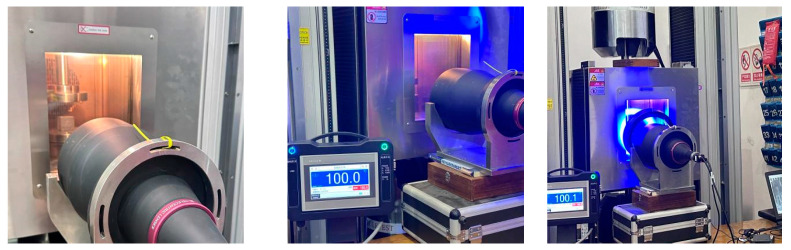
DIC experimental facility.

Conventional universal testing machines are insufficient for achieving the desired high-temperature environment conditions required for cement-based composite materials. Therefore, to meet the experimental requirements, specialized equipment was employed, as illustrated in Figure 3. A high-temperature environment box was installed within the pressure head of the testing machine to provide the necessary temperature conditions for the experiments.

A DNS-100 universal tester with a load capacity of 600 kN was used to simulate quasi-static loading through uniaxial compression at a loading rate of 1 mm/min. Throughout the test, DIC [26,27,28,29,30] was used to monitor the real-time full-field deformation of the samples. The image acquisition hardware consisted of a cold light source, BT-23120 telephoto lens, MV-EM510 M/C CCD camera, and bracket provided by Wilkesh Digital Image Technology Co., LTD. (Beijing, China). The telecentric lens had a magnification of 0.072, while the CCD had a resolution of 2456 pixels × 2058 pixels, totaling 5 million pixels. The image acquisition software, provided by Wilkesh Digital Image Technology Co., LTD. (Beijing, China), supported the experiment, with an image acquisition rate set at 6 frames/s. The acquired images of the entire loading process were processed using the 2D-DIC system of CSI Company (Beijing, China).

## 3. Test Methods

The high-temperature testing method employed in this study follows the same principles as the compressive strength testing of concrete under normal temperature conditions. The novelty lies in the treatment of the cement-based sample within a high-temperature environment chamber, maintaining the elevated temperature during the compression experiment. The entire testing process can be divided into the following two stages:

First stage: The cement-based sample is subjected to a temperature conditioning process. As concrete is a thermally inert material, the internal 3D printing lattice needs to reach a high-temperature state. To achieve this, the sample is preheated for a duration of 45 min, considering the curing age of the cement base, the desired test time, and relevant literature references. This preheating aims to establish an overall high-temperature boundary for the sample. Although the heating conditions in indoor physical tests may not precisely replicate those of a natural high-temperature environment, they provide insights into the general effects of temperature on the mechanical properties of concrete in an indoor testing setting. Second stage: The cement-based sample undergoes uniaxial compression testing within the high-temperature environment.

## 4. Results and Discussion

Uniaxial compression tests were conducted on different structures of 3D printed lattice-reinforced cement-based composites within a high-temperature environment. The samples were divided into three groups, each containing nine cement-based composite material samples with varying structures. The compression tests were performed at different ambient temperatures: room temperature, 50 °C, and 100 °C. Within each set of peak loads, the largest data group was selected for subsequent analysis of the stress–strain curve and strain cloud maps.

### 4.1. Stress–Strain Curve Analysis

**Figure 4:**
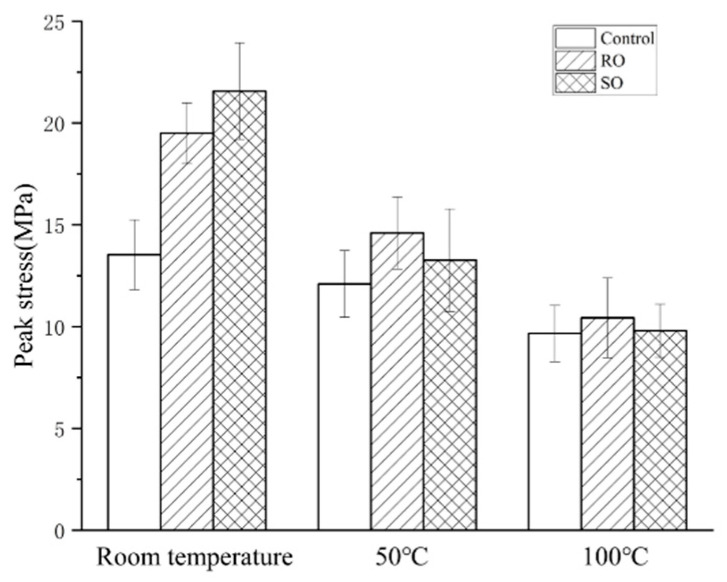
Peak stress and stress–strain curves of cement-based samples reinforced with different structures at various ambient temperatures.

In this experiment, the peak value refers to the maximum load sustained by the sample before failure and serves as a crucial indicator for evaluating the mechanical properties of 3D printed lattice-reinforced cement-based composites. As illustrated in Figure 4, the peak stress of the cement-based composite decreases with the increasing temperature. At an ambient temperature of 50 °C, the peak load of the non-lattice-reinforced cement-based composite is 89% of the value observed under normal temperature conditions. The peak load for the RO lattice-reinforced cement-based composite is 75% of the normal temperature environment, while the peak load for the SO lattice-reinforced cement-based composite is 62% of the maximum load that the material can withstand at room temperature.

**Figure 5:**
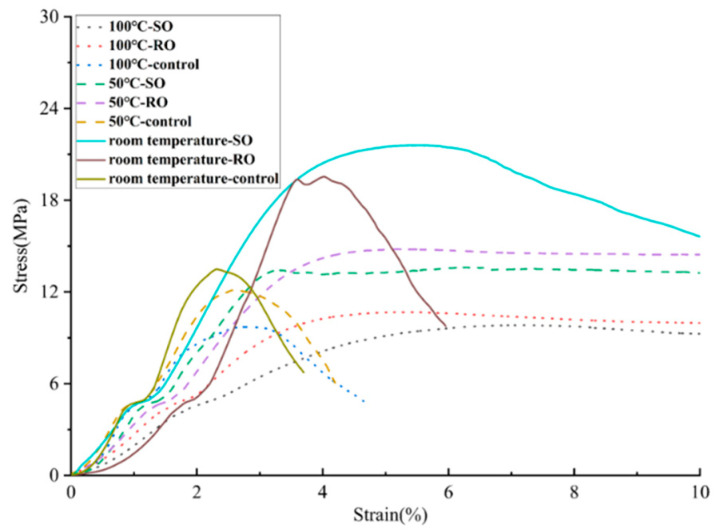
Stress–strain curve of compression test at high temperature.

As depicted in Figure 5, when the temperature reaches 100 °C, both the RO and SO lattice-reinforced cement-based composites experience a reduction in peak load of about 50%. In comparison, the plain cement-based composite without 3D printing exhibits a decrease in peak load of 72%. Due to the high temperature sensitivity of the internal 3D printed polymer lattice, the high-temperature mechanical properties of specimens with internal reinforcements decrease more. As the temperature increases, the internal polymer lattice gradually softens and loses its load-bearing capacity. Due to the softening of the internal reinforcement, the interface between the reinforcement and the cement matrix also gradually fails, ultimately leading to a significant decrease in the load-bearing capacity of such materials. These results indicate that the 3D printing reinforced cement-based composites demonstrate higher sensitivity to temperature changes. However, even with this temperature sensitivity, the peak load of the 3D printing reinforced cement-based composites remains superior to that of the plain cement-based composite samples.

### 4.2. DIC Analysis

**Figure 6:**
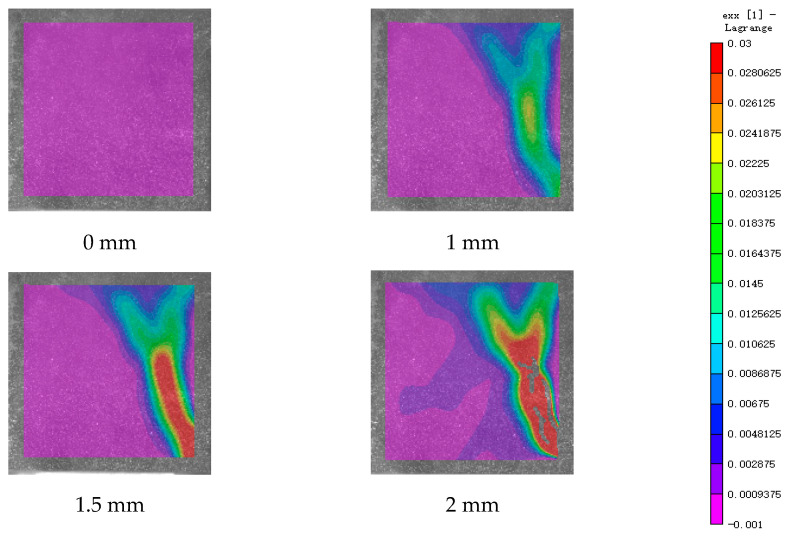
Strain cloud diagram of cement-based sample at ambient temperature.

**Figure 7:**
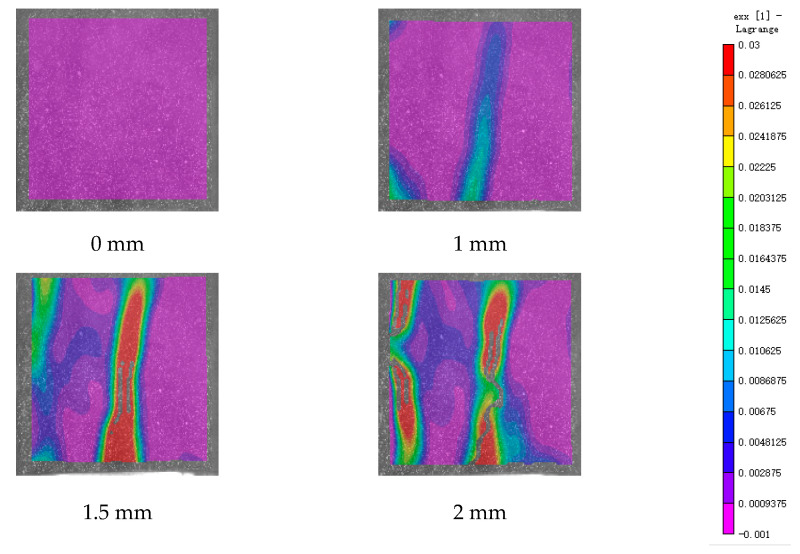
Strain cloud diagram of cement-based sample at 50 °C.

**Figure 8:**
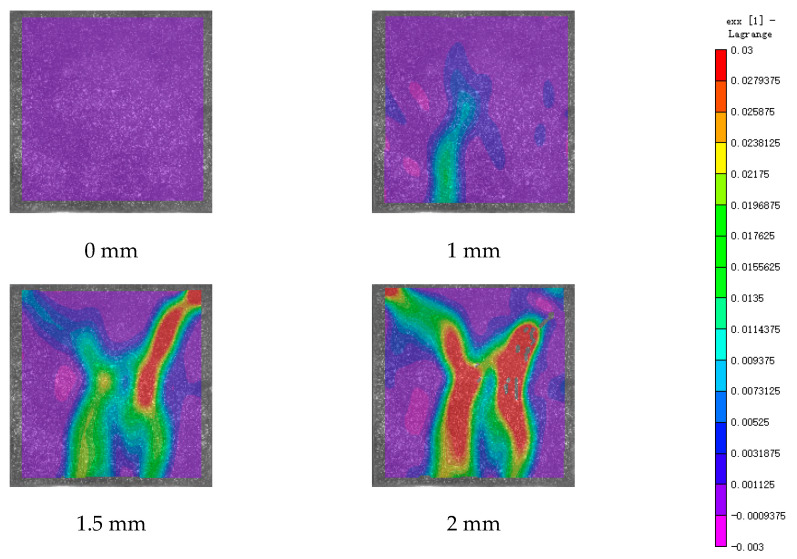
Strain cloud diagram of cement-based sample at 100 °C.

DIC technology mainly captures the natural texture or artificially created speckle patterns on the surface of the sample using industrial cameras. Subsequently, these acquired images undergo image processing algorithms to extract crucial information such as surface deformation during the test. In this study, VIC-2D software (V6) was employed to analyze and process the images obtained through DIC monitoring during the experiment. During the uniaxial compression experiment, the cement-based sample demonstrates transverse expansion and negative strain. In this scenario, the DIC results reveal notable distribution characteristics in the horizontal strain field of the sample, while the vertical strain field does not exhibit distinct features. Figure 6, Figure 7 and Figure 8 illustrate the strain evolution process of the cement-based samples at different temperatures under different displacements.

During the application of a compressive load, friction occurs at the contact interface between the indenter and the sample within the universal testing machine. This friction reduces the constraint on the sample in the edge region, making it susceptible to tension due to localized strains. As the loading progresses, when the tensile strain reaches the ultimate tensile strain of the cement matrix material, cracks begin to form in regions where tensile strain is concentrated. These cracks then propagate and expand with increasing load, ultimately resulting in structural failure through cracking. The deformation mechanism remains unaffected by the increase in ambient temperature, although the strain region of the cement-based sample expands.

**Figure 9:**
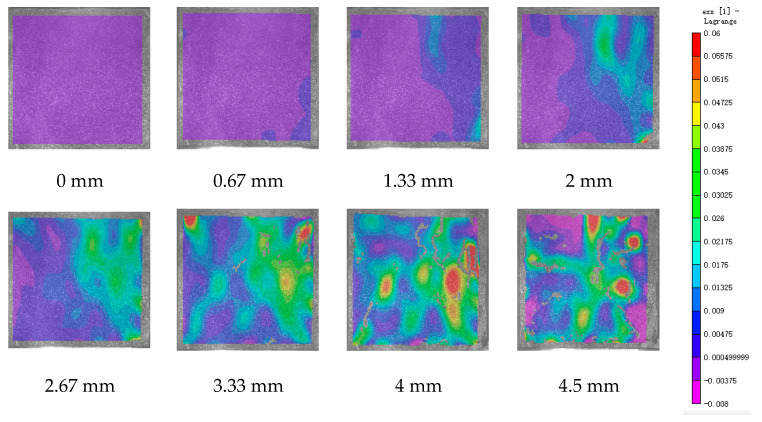
Strain cloud map of RO cement-based sample at room temperature.

**Figure 10:**
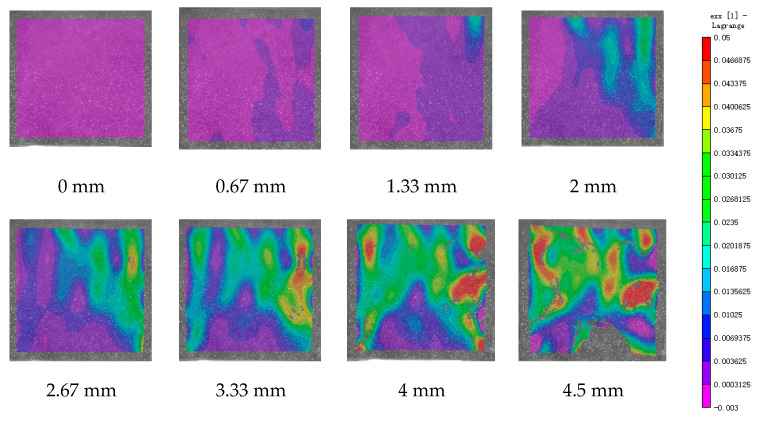
Strain cloud map of RO cement-based sample at 50 °C.

**Figure 11:**
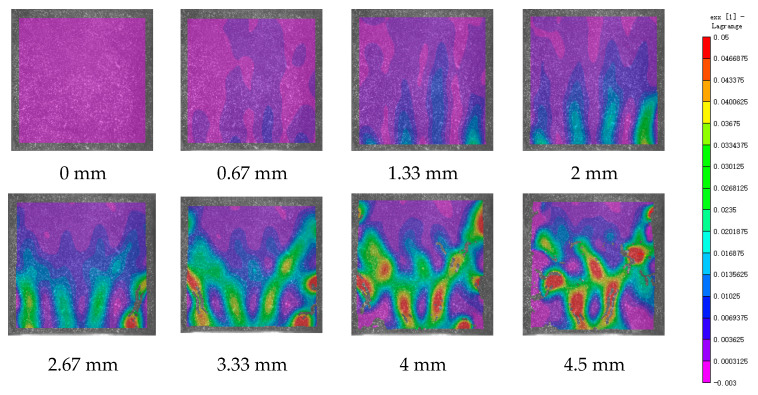
Strain cloud map of RO cement-based sample at 100 °C.

**Figure 12:**
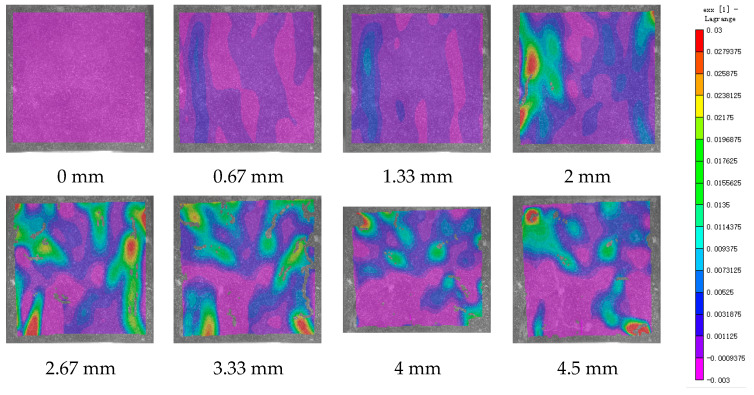
Strain cloud map of SO cement-based sample at room temperature.

**Figure 13:**
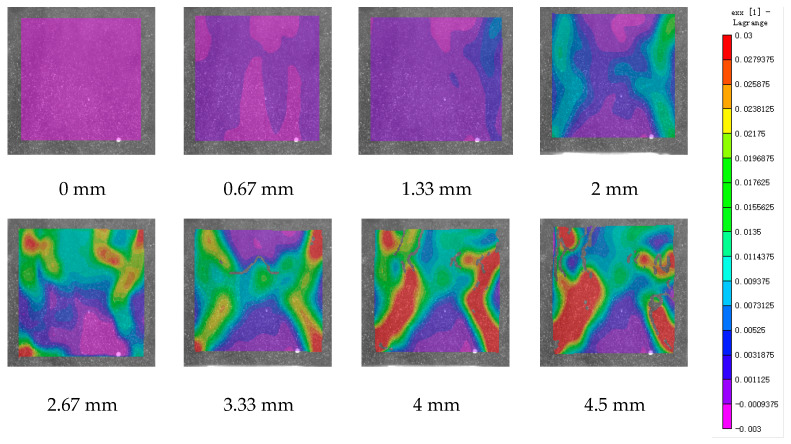
Strain cloud map of SO cement-based sample at 50 °C.

**Figure 14:**
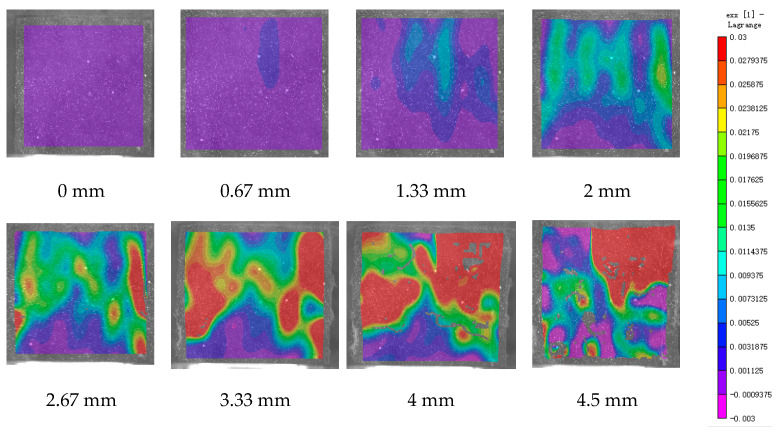
Strain cloud map of SO cement-based sample at 100 °C.

Figure 9, Figure 10, Figure 11, Figure 12, Figure 13 and Figure 14 present the strain cloud maps of RO and SO structural lattice-reinforced cement-based composites during uniaxial compression experiments at different ambient temperatures under different displacements.

Throughout the compression process, the sample undergoes a redistribution of strain, resulting from the structural adjustments and stress redistribution within the 3D printed polymer lattice. This characteristic plays a crucial role in dispersing the cracks that may form within the specimen under pressure, effectively preventing the development of significant cracks.

Nonetheless, as the ambient temperature rises, the limited heat resistance of the 3D printed polymer becomes evident. The expansion of the strain region observed in the sample significantly diminishes its compressive performance.

While the high-temperature environment can somewhat diminish the compressive mechanical properties of the material, it is still evident, in comparison to the normal temperature environment, that the 3D printed lattice plays a significant role in delaying the cracking of the cement matrix.

### 4.3. Fracture Morphology Analysis

**Figure 15:**
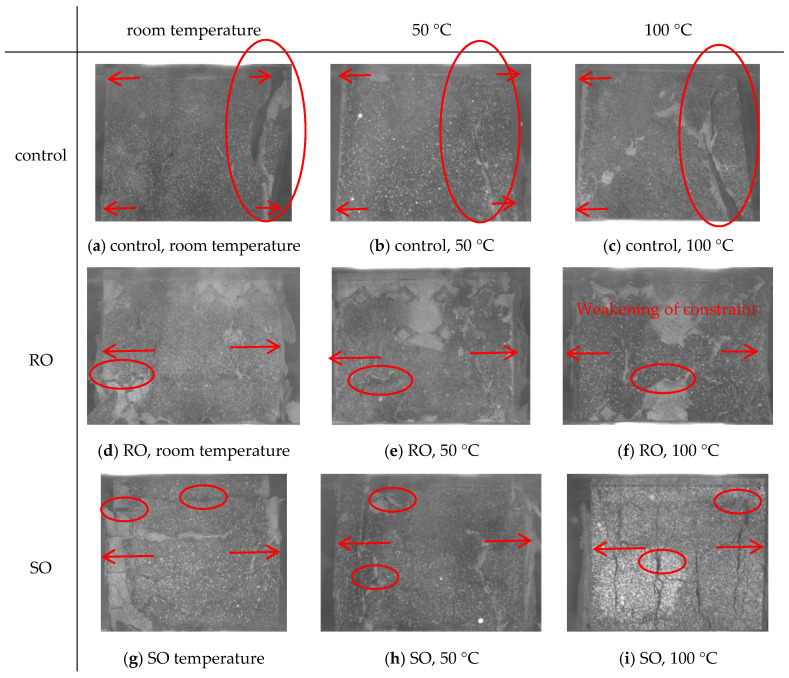
Morphology after failure of cement-based materials reinforced by different lattices at varying temperatures.

Figure 15 illustrates the morphology of the samples after failure under uniaxial compression loading. For the cement-based samples without lattice reinforcement (Figure 15a–c), the friction between the indenter of the testing machine and the upper and lower surfaces of the sample creates strong constraints on these surfaces, while the middle part of the sample experiences fewer constraints. As a result, the failure mode of the unreinforced cement-based sample is characterized by wider upper and lower surfaces in contact with the indenter, while the unconstrained middle part appears narrower, forming the shape depicted in the figure.

In contrast, for cement-based composites with RO and SO lattice reinforcement (Figure 15d–i), the 3D printed lattices significantly enhance the compressive strength of the cement-based materials. The strengthening mechanism of the 3D printing lattice on cement-based materials can be mainly attributed to the following factors:

(1) Restriction of cement matrix cracking: The 3D printed polymer lattice pillars effectively restrain the lateral deformation of the cement matrix, thereby significantly improving the compressive capacity of the cement-based composites.
(2) Prevention of cement pattern cracking: The lattice material bonds the various phases of the cementing material together, effectively controlling the generation and expansion of cracks within the sample and preventing the formation of large cracks. Consequently, the lattice material effectively fulfills its load-bearing role.

As depicted in Figure 15d–f, cement-based materials reinforced with an RO lattice exhibit a notable phenomenon of material stripping around the structure during the progression of uniaxial compression. In this process, the internal lattice of the sample effectively restrains the cracking of the matrix, resulting in a delayed structural failure compared to the cement-based material without the lattice. However, as the temperature increases, the bearing capacity of the 3D printing lattice decreases due to the influence of temperature, causing a weakening of the constraint effect on the cement matrix. Figure 15g–i demonstrates that cement-based samples reinforced with SO lattice display similar deformation characteristics.

## 5. Conclusions

This study aimed to evaluate the compressive properties of cement-based composites reinforced with different lattice structures under varying ambient temperatures. The effects of a high-temperature environment on 3D printed lattice-reinforced cement-based materials were analyzed using peak stress, stress–strain curves, and DIC image analysis. The findings indicate that the high-temperature environment leads to a reduction in the material’s bearing capacity. Moreover, the 3D printed lattice-reinforced cement-based composites exhibit higher temperature sensitivity compared to the cement-based composites without polymer lattice reinforcement. However, the temperature variations do not alter the strain evolution law of the composite material. The incorporation of the lattice structure significantly inhibits the transverse deformation of the cement-based composite material under uniaxial compression loads. It redistributes the internal stress within the matrix, delays crack expansion, and ultimately improves the compressive bearing capacity of the cement-based composite material.

## Figures and Tables

**Table 1:** Design parameters of 3D printing lattice structures.

Serial Number	Lattice Cell	Cellular Structure	Cell Parameter	CAD Structure
1	RO	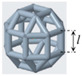	l = 4.14 mm d = 1.659 mm	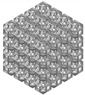
2	SO	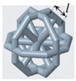	l = 4.14 mm d = 0.979 mm	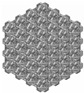

**Table 2:** Mineral composition of ordinary Portland cement PO42.5.

Component composition	C_3_S	C_2_S	C_4_AF	C_3_A	F-MgO	F-CaO
Content (%)	60.5	18.1	8.9	7.4	1.8	0.9

**Table 3:** Performance index of water-reducing agent.

Air Content	3.4%
Moisture content	1.7%
pH value (23 °C)	8.0 ± 1.0
Volume density	600 ± 100 g
Sodium sulfate content	0.79% m^3^
Cl^−^ content	0.049%
Total alkalinity	0.77%
Powder color	white powder
Water reduction rate of cement mortar	≥30%
Recommended dosage	0.15–0.3

**Table 4:** Composition of sand.

SO_2_	>98%	Loss on ignition	<0.47%
Moisture content	≤0.18%	Cl^−^ content	≤0.007%
Sediment content	≤0.18%	Other mineral content	≤0.002%

**Table 5:** Cement-based composite material mix ratio.

Cement	Fly Ash	Standard Sand (Particle Size of <0.63 mm)	Water-Reducing Agent	Water
410 g	40 g	1350 g	1.35 g	280 g

## Data Availability

The original contributions presented in this study are included in the article. Further inquiries can be directed to the corresponding author.
